# Supplementary figures and images for: Molecular cytogenetic mapping of Secale strictum introgressions in a perennial tetraploid rye and its diploid progenitor
Source: PLoS One. 2026 May 13;21(5):e0349207. doi: 10.1371/journal.pone.0349207 (PMC13170958; doi:10.1371/journal.pone.0349207)

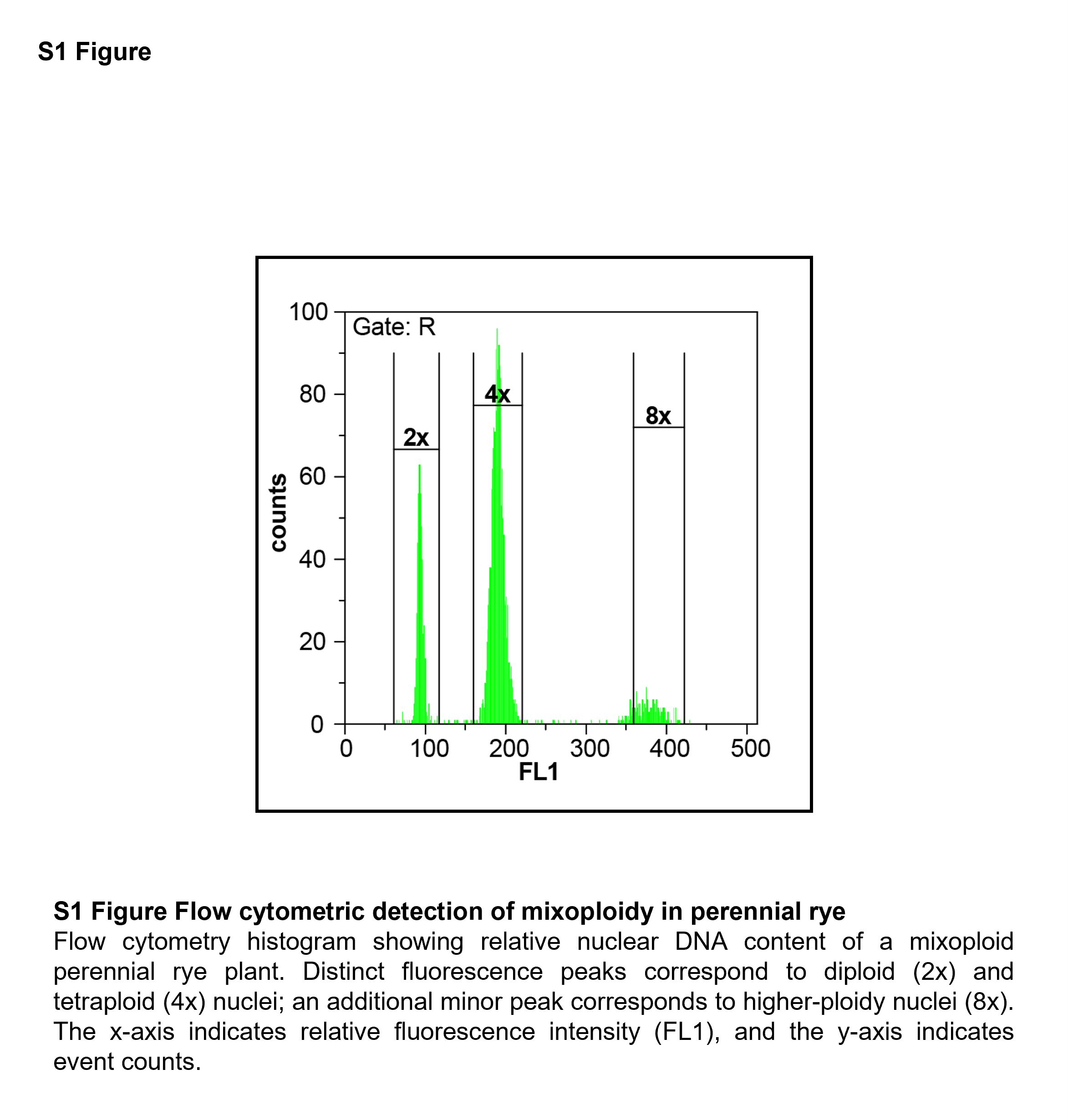

Supplement: S1 Fig — Flow cytometry histogram showing relative nuclear DNA content of a mixoploid perennial rye plant. Distinct fluorescence peaks correspond to diploid (2x) and tetraploid (4x) nuclei; an additional minor peak corresponds to higher-ploidy nuclei (8x). The x-axis indicates relative fluorescence intensity (FL1), and the y-axis indicates event counts. (TIF) [file pone.0349207.s001.tif]

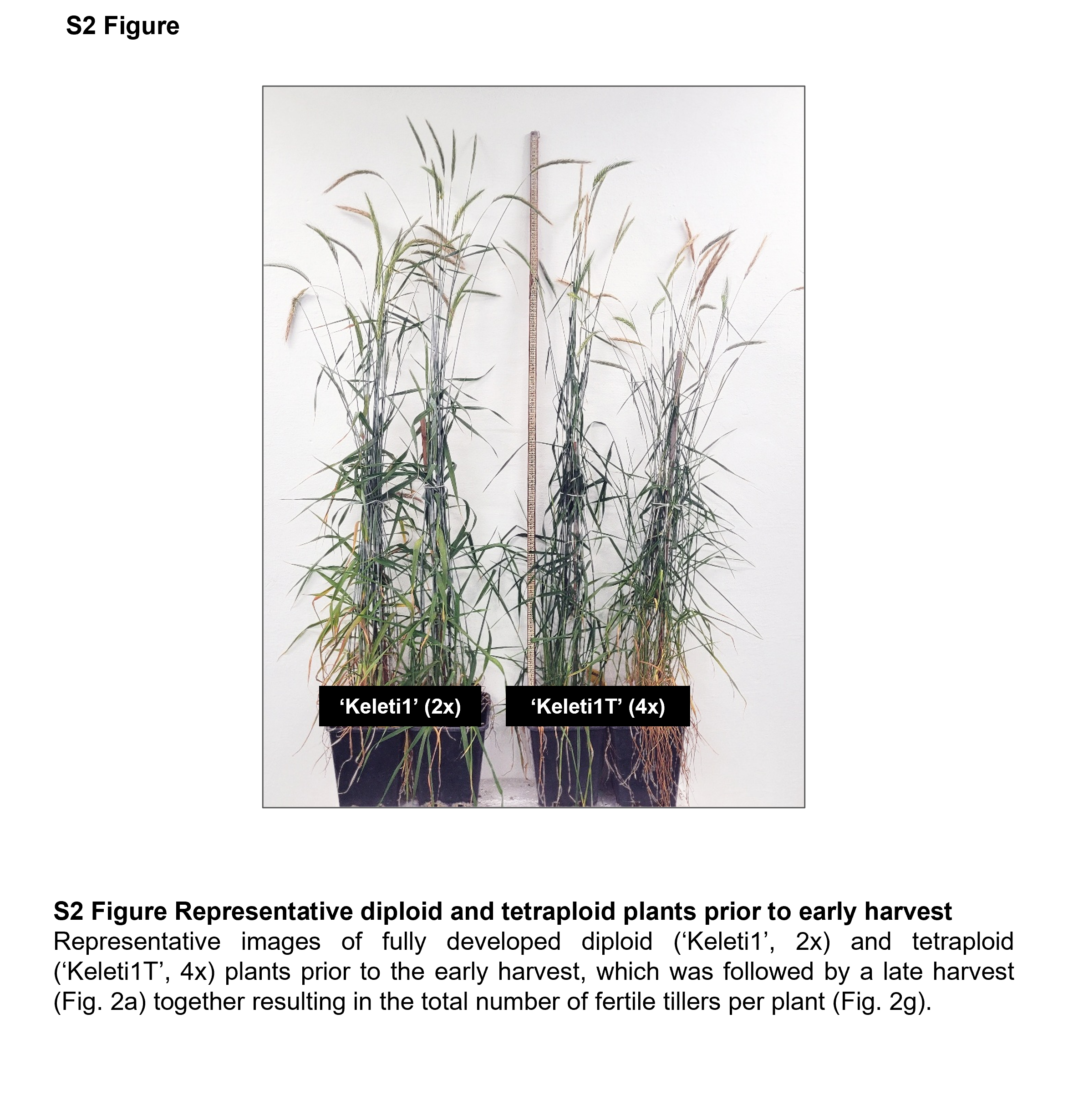

Supplement: S2 Fig — Representative images of fully developed diploid (‘Keleti1’, 2x) and tetraploid (‘Keleti1T’, 4x) plants prior to the early harvest, which was followed by a late harvest (Fig. 2A) together resulting in the total number of fertile tillers per plant (Fig. 2G). (TIF) [file pone.0349207.s002.tif]

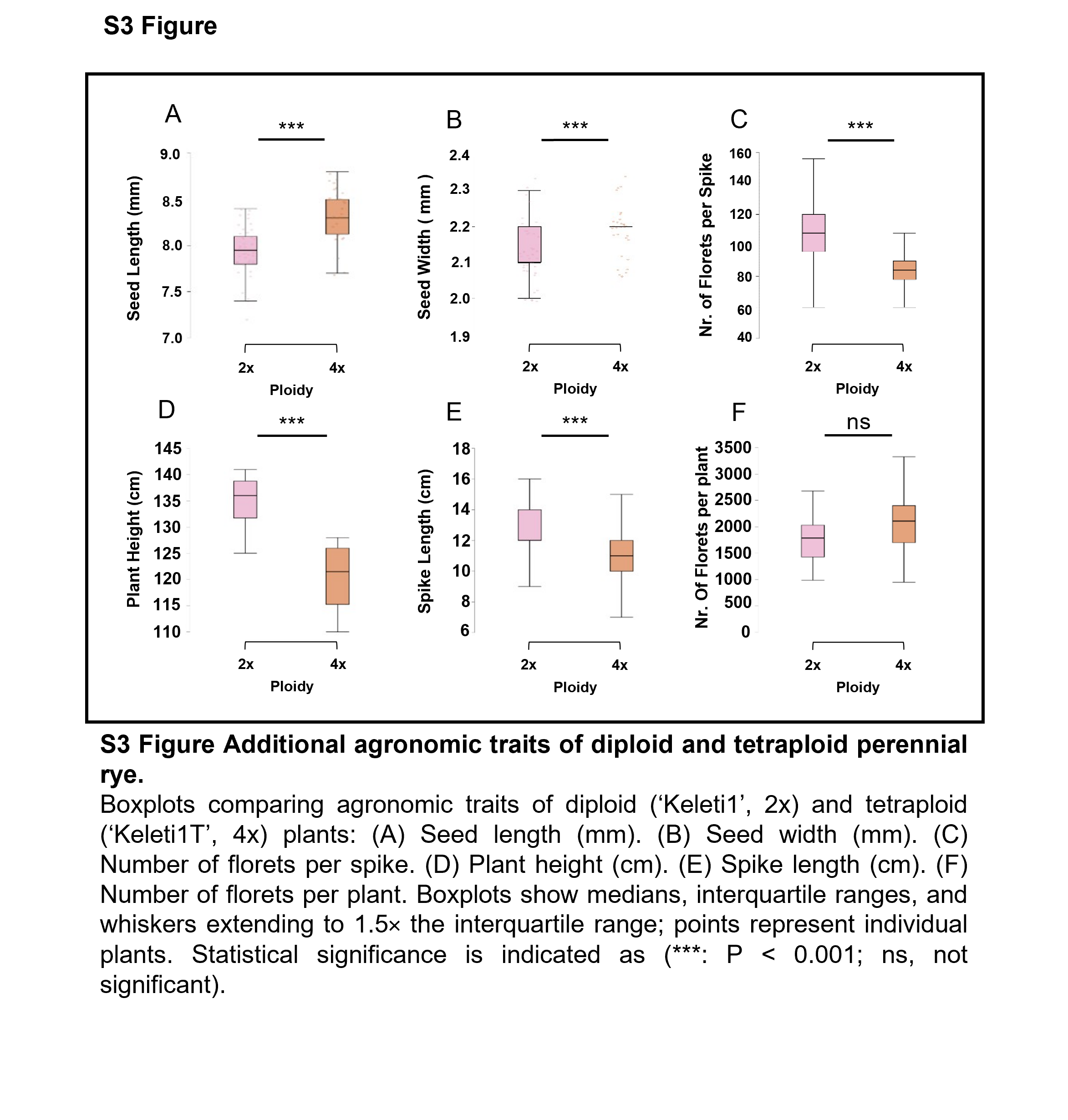

Supplement: S3 Fig — Boxplots comparing agronomic traits of diploid (‘Keleti1’, 2x) and tetraploid (‘Keleti1T’, 4x) plants: (A) Seed length (mm). (B) Seed width (mm). (C) Number of florets per spike. (D) Plant height (cm). (E) Spike length (cm). (F) Number of florets per plant. Boxplots show medians, interquartile ranges, and whiskers extending to 1.5 × the interquartile range; points represent individual plants. Statistical significance is indicated as (***: P < 0.001; ns, not significant). (TIF) [file pone.0349207.s003.tif]

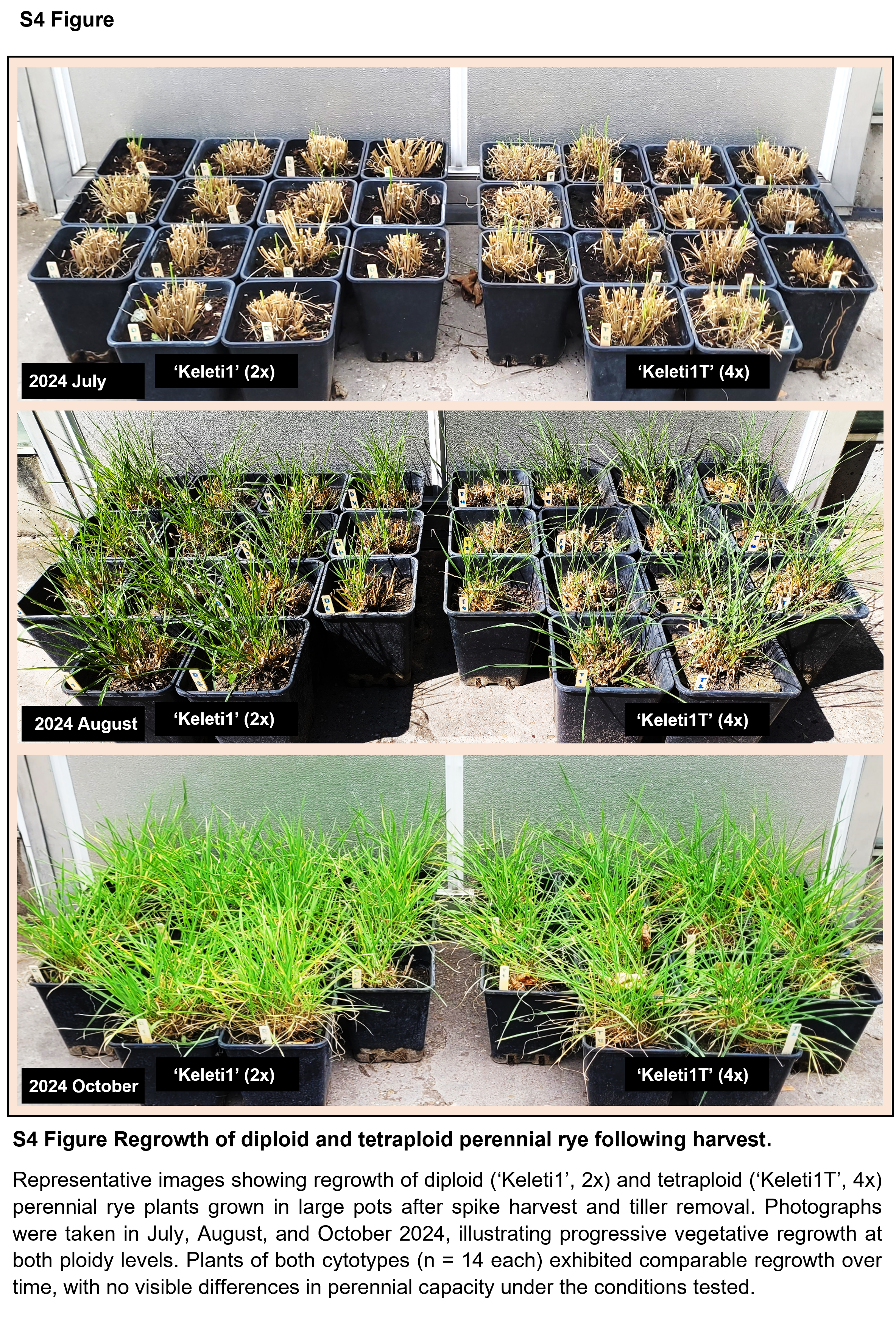

Supplement: S4 Fig — Representative images showing regrowth of diploid (‘Keleti1’, 2x) and tetraploid (‘Keleti1T’, 4x) perennial rye plants grown in large pots after spike harvest and tiller removal. Photographs were taken in July, August, and October 2024, illustrating progressive vegetative regrowth at both ploidy levels. Plants of both cytotypes (n = 14 each) exhibited comparable regrowth over time, with no visible differences in perennial capacity under the conditions tested. (TIF) [file pone.0349207.s004.tif]

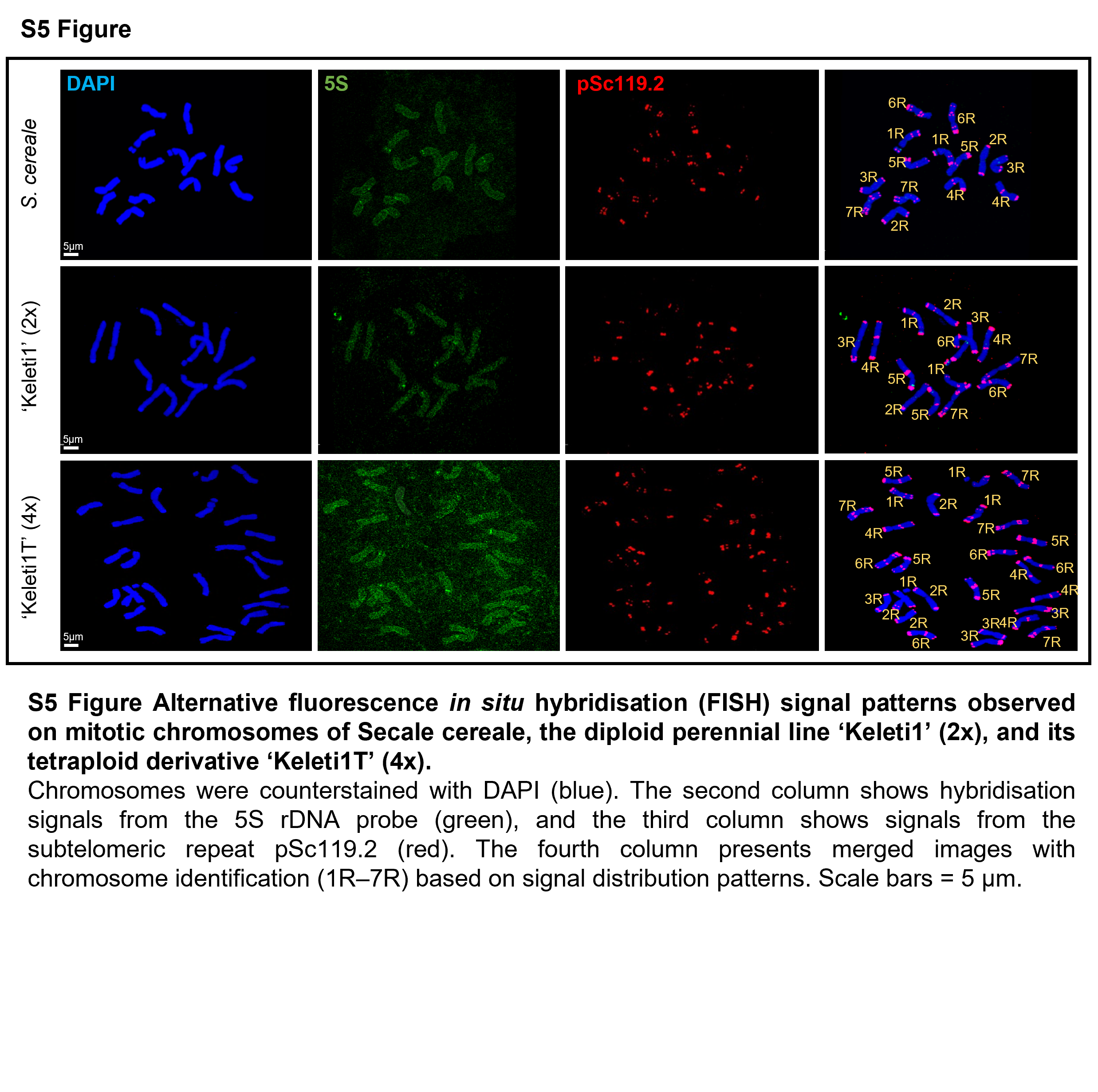

Supplement: S5 Fig — Chromosomes were counterstained with DAPI (blue). The second column shows hybridisation signals from the 5S rDNA probe (green), and the third column shows signals from the subtelomeric repeat pSc119.2 (red). The fourth column presents merged images with chromosome identification (1R–7R) based on signal distribution patterns. Scale bars = 5 μm. (TIF) [file pone.0349207.s005.tif]

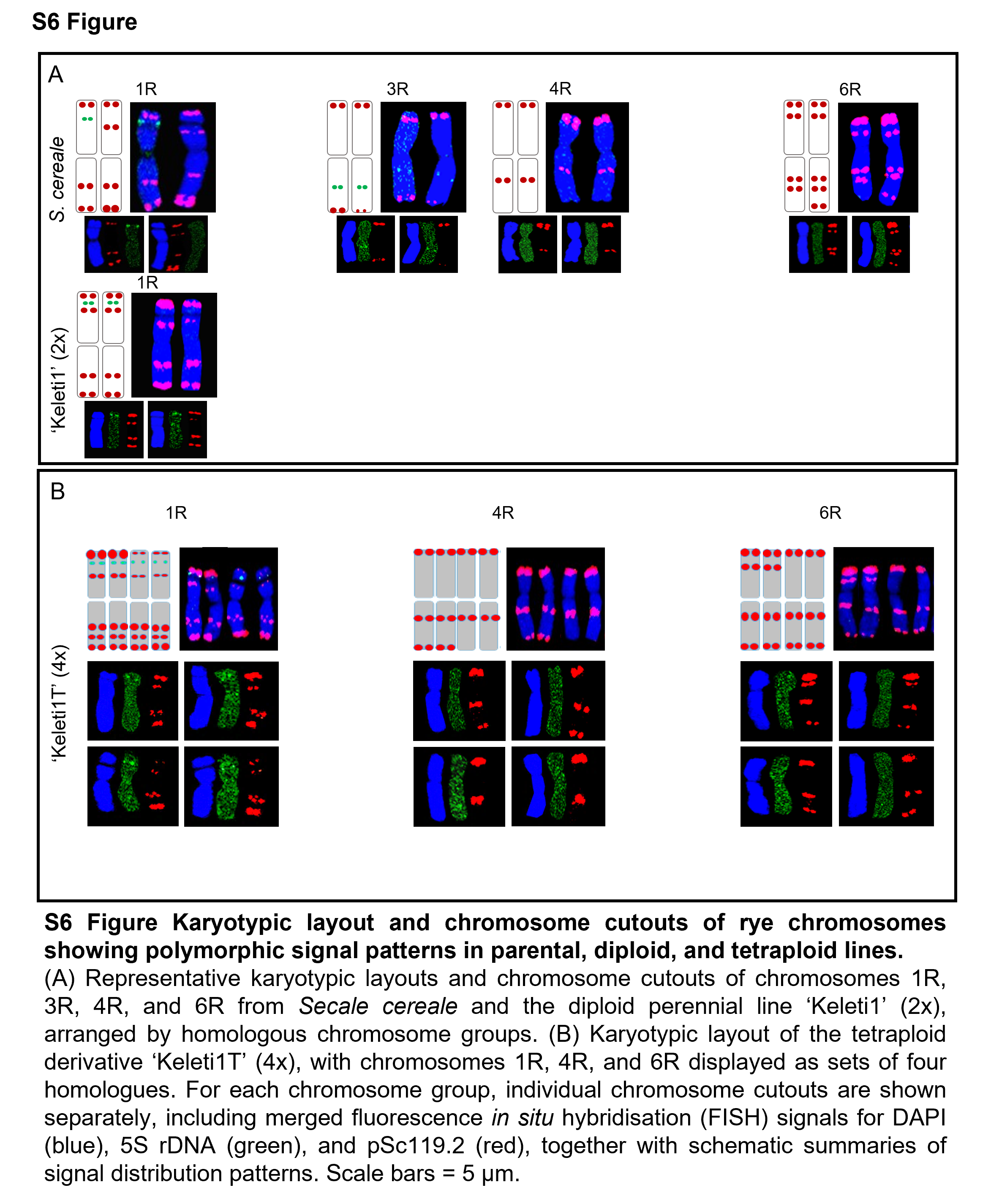

Supplement: S6 Fig — (A) Representative karyotypic layouts and chromosome cutouts of chromosomes 1R, 3R, 4R, and 6R from Secale cereale and the diploid perennial line ‘Keleti1’ (2x), arranged by homologous chromosome groups. (B) Karyotypic layout of the tetraploid derivative ‘Keleti1T’ (4x), with chromosomes 1R, 4R, and 6R displayed as sets of four homologues. For each chromosome group, individual chromosome cutouts are shown separately, including merged fluorescence in situ hybridisation (FISH) signals for DAPI (blue), 5S rDNA (green), and pSc119.2 (red), together with schematic summaries of signal distribution patterns. Scale bars = 5 μm. (TIF) [file pone.0349207.s006.tif]
