# Supplementary material for: Molecular cytogenetic mapping of Secale strictum introgressions in a perennial tetraploid rye and its diploid progenitor
Source: PLoS One. 2026 May 13;21(5):e0349207. doi: 10.1371/journal.pone.0349207 (PMC13170958; doi:10.1371/journal.pone.0349207)
Supplement: Attachment — Submitted filename: Comments of manuscript PONE-D-26-04864.docx [file pone.0349207.s008.docx]

Comments of manuscript PONE-D-26-04864

Employing the newly developed 'Keleti1' line and its tetraploid derivative 'Keleti1T', this study examines the impact of genome duplication on agronomic traits and chromosomal architecture through integrated phenotypic and FISH-based cytogenetic analyses. The work is well-conceived and data-rich, providing important theoretical insights and practical cytogenetic tools for perennial rye improvement. The manuscript is of solid quality overall, though certain details and lines of reasoning could be further refined. The manuscript can be accepted after minor revision as the following comments:

1. In the comparison of agronomic traits and karyotype analysis between diploid and tetraploid plants, only 14 individuals were examined per cytotype. This relatively small sample size may affect the reliability of the comparative results. It is recommended that this limitation be explicitly acknowledged in the Discussion section.

2. There is an inconsistency between the phenotypic data and the representative plant images, raising a question about the use of terminology related to tillering. In the Results section, the statistical data indicate that the mean number of fertile tillers (spikes) per plant exceeds 17 in diploids and 24 in tetraploids. However, in the plant images shown in Figure 2a, the number of visible fertile tillers (effective tiller?) is considerably lower than these statistical values. This discrepancy suggests that the statistical data may be more representative of the total tiller rather than the fertile tiller. Is there a potential confusion between these two concepts?

3. In the evaluation of perennial growth habit, the diploid 'Keleti1' was compared in the field with its perennial parent *S. strictum* for regrowth rate (Figure 3). However, in the semi-outdoor pot experiment assessing the perennial habit of the tetraploid, it was only compared with the diploid 'Keleti1', lacking a direct comparison with the original perennial parent, *S. strictum*. While not a critical flaw, including this comparison would allow for a more comprehensive assessment of the impact of polyploidization on perennial growth habit.

4. Figure 3 compares the regrowth performance of the diploid 'Keleti1' and its parental species *S. strictum*. To enhance figure clarity, it is recommended that the two groups be clearly distinguished and labeled, facilitating direct visual comparison and interpretation by readers.

5. The Results section indicates substantial variation in seed number per plant among tetraploid individuals, with some reaching relatively high values. Concurrently, karyotypic differences (e.g., in pSc119.2 signal distribution) were observed among these tetraploid plants. It is therefore recommended to further analyze the cytological characteristics (e.g., specific FISH karyotypes) of these "high-value" tetraploid individuals to explore whether specific chromosomal karyotypes are associated with higher seed numbers. Correlating the phenotypic data with cytological data would enhance the logical coherence and scientific depth of the manuscript.

6. In the legends of Figure 2 and Supplementary Figure S2, it is recommended that significance levels be clearly indicated (e.g., **: *P* < 0.01; ***: *P* < 0.001) to improve result transparency and interpretability.

7. Please carefully check the formatting of the references. For instance, only the first word of article titles should be capitalized (see references 3, 6, 8, and 9, among others).
